# Exploring Plasma Proteome Thermal Stability in Peripheral Arterial Disease: Biophysical Findings Under Cilostazol Therapy

**DOI:** 10.3390/ph18060886

**Published:** 2025-06-13

**Authors:** Dorottya Szabó, László Benkő, Dénes Lőrinczy

**Affiliations:** 1 Department of Vascular Surgery, Medical School, University of Pécs, Pécs Ifjúság Str. 13, H-7624 Pecs, Hungary; dr.szabo.dorottya@pte.hu (D.S.); benko.laszlo@pte.hu (L.B.); 2 Department of Biophysics, Medical School, University of Pécs, Pécs Szigeti Str. 12, H-7624 Pecs, Hungary

**Keywords:** cilostazol, peripheral arterial disease, intermittent claudication, DSC, human plasma

## Abstract

**Introduction**: Intermittent claudication, an early symptom of peripheral artery disease, can be treated by cilostazol to alleviate symptoms and improve walking distance. Our previous investigation focused on cilostazol-induced alterations in the thermodynamic properties of plasma, utilizing differential scanning calorimetry (DSC) as a potential monitoring tool. The current proof-of-concept study aimed to enhance the interpretation of DSC data through deconvolution techniques, specifically examining protein transitions within the plasma proteome during cilostazol therapy. **Results**: Notable differences in thermal unfolding profiles were found between cilostazol-treated patients and healthy controls. The fibrinogen-associated transition exhibited a downward shift in denaturation temperature and decreased enthalpy by the third month. The albumin-related transition shifted to higher temperatures, accompanied by lower enthalpy. Transitions associated with globulins showed changes in thermal stability, while the transferrin-related peak demonstrated increased structural rigidity in treated patients compared to controls. **Discussion**: These observations suggest that cilostazol induces systemic changes in the thermodynamic behavior of plasma proteins. DSC, when combined with deconvolution methods, presents a promising approach for detecting subtle, therapy-related alterations in plasma protein stability. **Materials and methods**: Ten patients (median age: 58.6 years) received 100 milligrams of cilostazol twice daily. Blood samples were collected at the baseline and after 2 weeks, 1 month, 2 months, and 3 months of therapy. Walking distances were also assessed. The DSC curves were retrieved from the thermal analysis investigated by deconvolution mathematical methods. **Conclusions**: Although the exact functional consequences remain unclear, the observed biophysical changes may reflect broader molecular adaptations involving protein–protein interactions, post-translational modifications, or acute phase response elements.

## 1. Introduction

Intermittent claudication (IC) is a definitive symptom at the early stage of peripheral artery disease (PAD), a circulatory problem of the lower extremities that causes reduced blood flow through the arteries. IC manifests as exertional pain in the calf, thigh, and/or buttock that does not begin at rest, does not resolve during walking activity, and resolves within 10 min of rest [1]. The prevalence of IC in the Edinburgh Artery Study was 4.5% and equally common in both sexes [2].

The treatment options for IC include conservative therapy and surgical or endovascular treatment. Conservative treatment of IC consists of risk factor management (weight loss, healthy diet, smoking cessation), exercise therapy, and the best medical treatment (single antiplatelet therapy, lipid-lowering, antihypertensive, and antidiabetic drugs) [3,4].

Cilostazol is a selective inhibitor of phosphodiesterase III, which is frequently used in conservative treatment of patients with slight or moderate IC to reduce symptoms and improve walking distance [3,4]. Cilostazol increases intracellular cAMP levels, blocks platelet aggregation, and promotes arterial vasodilation [5]. In the metabolism of cilostazol, CYP3A4, CYP2C19, and CYP3A5 are primarily involved [6].

According to the recent guideline of European Society for Vascular Surgery, “for patients with lifestyle limiting intermittent claudication who adhere to best medical treatment including exercise therapy, cilostazol or naftidrofuryl oxalate should be considered, to improve walking distance, but treatment should be stopped after three to six months of therapy if no improvement has been noted.” (Class IIa, Level A) [3]. The ACC/AHA/AACVPR/APMA/ABC/SCAI/SVM/SVN/SVS/SIR/VESS Guideline for the Management of Lower Extremity Peripheral Artery Disease states that “in patients with claudication, cilostazol is recommended to improve leg symptoms and increase walking distance.” (Class I, Level A). This guideline also highlights that assessing patient tolerance of cilostazol may be valuable at 2 to 4 weeks and evaluating benefits within 3 to 6 months to determine whether long-term therapy will be beneficial [4]. However, it is essential to emphasize that, according to the European Medicines Agency, cilostazol is labeled as contraindicated in patients with a history of congestive heart failure, unstable angina, severe tachyarrhythmia or with any known predisposition of bleeding. It also should not be given to patients who have had myocardial infarction or a coronary intervention within the last six months. Severe renal impairment (creatinine clearance of ≤25 mL/min), moderate or severe hepatic impairment, and taking concomitantly two or more additional antiplatelet or anticoagulant agents are contraindications as well [7]. Cilostazol is 95–98% protein bound, primarily to albumin. The dehydro metabolite is 97.4% protein bound, and the 4″-trans-hydroxy metabolite is 66% [8]. Therefore, monitoring the changing thermodynamic parameters of the plasma of patients taking cilostazol can be relevant to monitoring cilostazol’s treatment efficacy, especially in cases where cilostazol is ineffective within the 3-month treatment period.

In our previous study, we aimed to explore other mechanisms by which cilostazol may exert its effect. We investigated cilostazol’s impact on the human plasma thermodynamic properties in PAD patients over a 3-month treatment period. We focused on developing the application of the differential scanning calorimetry (DSC) approach as a new monitoring method. Thermal transitions such as protein denaturation can be detected by this technique to assess changes in human plasma’s thermodynamic stability and structural properties. We hypothesized that alterations in the DSC profiles of human plasma would correlate with increased walking distance induced by cilostazol.

Biological samples can be decomposed into main and well-known components using the deconvolution method, primarily from the summation of DSC recordings of human plasma [9]. Plasma is a complex protein mixture. Therefore, it contains, from a thermal point of view, different “thermal domains” that can be assigned to the various compounds [10]. It means that a DSC scan can be decomposed into a sum of Gaussian curves; that way, their total area is nearly the same as the experimental curve one, within a reasonable error (<1%). Checking the contribution of the different protein compounds to the total calorimetric enthalpy, we can follow the effect of medical treatments compared to the healthy control.

Therefore, our recent aim was to measure the human plasma of patients with IC taking cilostazol using deconvolution. This study is a feasibility study. The DSC curves were retrieved from the thermal analysis investigated by deconvolution mathematical methods.

## 2. Results

**Figure 1:**
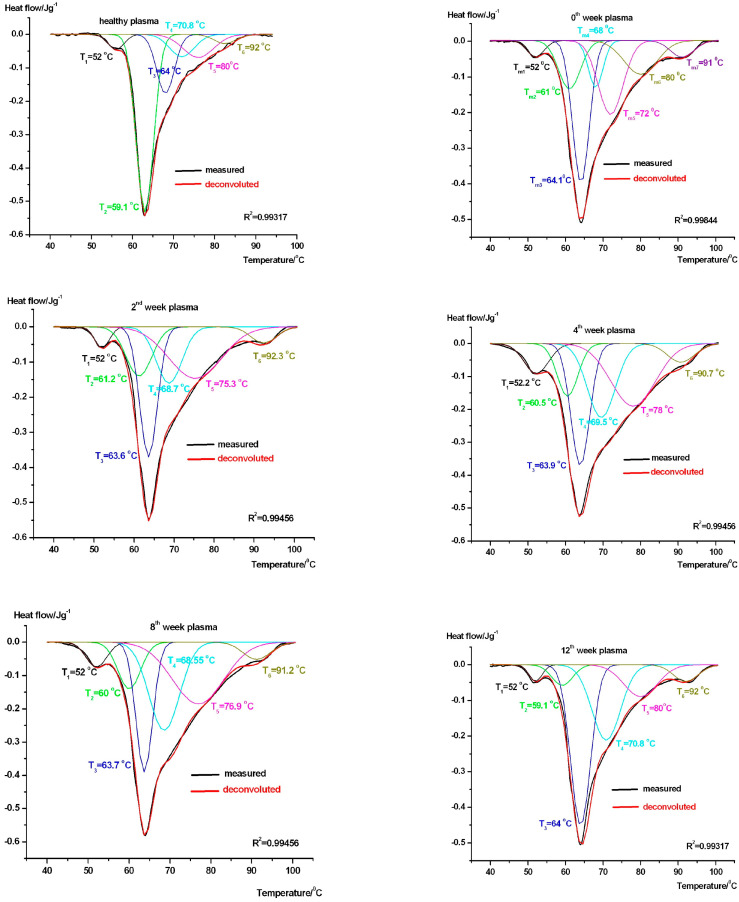
Average measured and deconvoluted denaturation scans of healthy control and patients after 0–2–4–8–12 weeks treatment (endotherm deflection is downwards).

We have plotted the averages of our measurements in Figure 1. The deconvolution procedure was based on literature data published by different research groups [10,11,12,13,14,15,16,17]. According to these, the following melting temperatures (*T_m_*) can be assigned to the most important plasma proteins: ~51 °C fibrinogen, ~62 °C unligated human serum albumin (HSA), and haptoglobulin, ~71 °C IgG, IgA and IgM with decreasing weight, as well as ~82 °C IgG and transferrin.

We have tried to achieve the best fitting to the experimental curves; this way, we obtained six decompositions. The relevant thermodynamic parameters are collected in Table 1.

**Figure 2:**
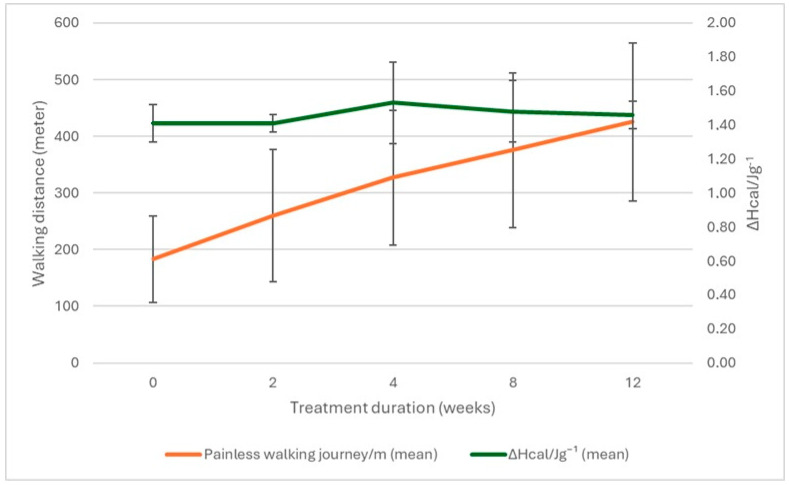
Walking distance and total calorimetric enthalpy as a function of treatment’s time. Large error bars reflect the exploratory nature and small sample size of this proof-of-concept investigation.

The patients’ walking distance increased during the examined period. The calorimetric enthalpy increased between weeks 2 and 4 and then slowly decreased, but it did not reach the starting point by the 12th week, as shown in Figure 2.

## 3. Discussion

The most common proteins in blood plasma are fibrinogens (that affect clotting) ~4%, albumins ~60%, and globulins ~36%. The figure and table clearly show the notable difference between healthy control and treated patient samples’ thermal denaturation (based on the DSC curves’ running and the thermal data). *T*_1_ stands for fibrinogen. It is higher in healthy samples than in our patients, where it fluctuates ~52 °C. The drop by 2 °C after one week of treatment should be the effect of adaptation to the treatment. This protein has a definite but relatively small contribution to the overall enthalpy at the 12th week of therapy, which might be a sign of the treatment’s side effects. It is commonly accepted that *T*_2_ could be assigned to the unfolding of albumin ~62 °C [10,11]. Its enthalpy contribution during unfolding is the highest because it is ~60% of the total protein content. It is clear the albumin contribution to the total enthalpy is considerably higher for healthy controls, as compared to the ones receiving the therapy. Our patients produced detectably smaller enthalpy contribution in this temperature range. It is probably the separate unfolding of haptoglobin [11], which is usually totally overlapped by albumin. *T*_3_, *T*_4_, and *T*_5_ unfolding peaks can be attributed to the globulins (IgG, IgA, and IgM), and *T*_6_ is the transferrin denaturation in the case of healthy control [11]. In our patients, the T3 transition corresponds to the albumin denaturation, occurring at ~64 °C instead of the usual 62 °C but with a smaller enthalpy contribution. This may represent a decrease in the amount of albumin plus a more rigid protein structure. Possible causes of a drop in patients’ albumin include liver failure, kidney disease, heart failure, gastrointestinal conditions, and malnutrition [18]. In treated patients, *T*_4_ and *T*_5_ show an alteration in the thermal stability of IgG and IgM, while *T*_6_ exhibits a more rigid transferrin structure [11,13].

Interestingly, the fibrinogen-associated thermal transition (*T*_1_) displayed a downward shift in denaturation temperature (~52 °C) with reduced enthalpy contribution following cilostazol treatment, particularly after 12 weeks. This suggests potential structural modification or decreased functional integrity, possibly linked to cilostazol’s systemic effects rather than direct interaction with fibrinogen, as no direct evidence exists for cilostazol-induced fibrinogen alteration [19]. However, studies have shown post-translational modifications in fibrinogen may affect its stability [20], and cilostazol’s anti-inflammatory action [21,22] could indirectly influence fibrinogen dynamics.

Additionally, we observed that the typical albumin-associated transition (*T*_2_), prominent in healthy controls (~62 °C), was notably attenuated in patients. This could be due to a combination of decreased albumin levels and altered binding or unfolding behavior, with haptoglobin signals becoming more pronounced in the same thermal region, suggesting a shift in plasma protein dynamics. Interestingly, the albumin-associated transition in patients (*T*_3_) was shifted to ~64 °C with a lower enthalpy, implying a potential increase in protein rigidity or post-translational modifications influencing albumin stability under cilostazol therapy. According to the cited authors, cilostazol can cause chemical denaturation, which might affect thermal denaturation [23], but drug binding to albumin might play a role. These findings might support our theory regarding the effect of cilostazol therapy, which has not been examined yet.

The globulin-related transitions (*T*_4_ and *T*_5_) displayed altered thermal stability, suggesting that cilostazol might also influence immune-related plasma proteins such as IgG and IgM.

Moreover, the transferrin-associated peak (*T*_6_) in treated patients exhibited signs of increased structural rigidity compared to controls. These findings might align with the following reports: according to the hypothesis of Kibel et al., transferrin plays an important role in atherosclerosis and in the etiology of oxidative stress in atherogenesis due to its antioxidant properties [24]. Furthermore, transferrin is an important clotting regulator and adjuster in maintaining coagulation balance because it modifies the coagulation cascade [25].

Taken together, these findings suggest that cilostazol modulates not only the functional capacity of blood vessels and platelets but also induces systemic changes in the plasma proteome’s thermodynamic behavior. While the functional implications of these biophysical modifications are yet to be fully elucidated, they could be indicative of broader molecular adaptations to cilostazol, including shifts in protein–protein interactions, post-translational modifications, or acute phase response elements.

These findings suggest that cilostazol therapy is associated with systemic biophysical changes in the plasma proteome, which could accompany its known pharmacological actions, such as antiplatelet and vasodilatory effects. Furthermore, our study demonstrates that DSC, combined with deconvolution, is a promising technique for detecting subtle, treatment-related changes in plasma protein stability.

It is essential to mention the limitations of the study. We take our work as a preliminary step because the application of DSC in this field is a new trend. Therefore, the sample size is small, and the follow-up time is short. We had to face many hindering factors, such as the willingness of patients to cooperate in a long-term program, remote residence, obstacles to the necessary sampling time, or dropping out of the study due to surgery or other health impairments requiring stopping taking cilostazol. Another crucial, in thermal analysis, wildly accepted factor is that when differences in denaturation temperatures are higher than 1–2 °C, that cannot be the consequence of the instrument’s sensitivity (in the case of temperature, it is in ±0.05 °C) or the reproducibility. Due to these reasons, applying any statistical analysis at this point would not be valid. Due to the small sample size, the error bars are also large in Figure 2.

## 4. Materials and Methods

Our preliminary study included ten patients with IC (five men and five women, median age: 58.6 years). We applied the following inclusion criteria: age between 40 and 75, walking distance less than 400 m (equal to IIa stage according to Fontaine classification), and no pedal pulses. Exclusion criteria were the presence of ischemic heart disease, chronic kidney failure, and limb-threatening ischemia.

The observation time was 3 months, except for one female patient. In that case, the patient had an irreversible ischemic injury in the right foot. Therefore, we had to perform a femoral amputation on day 78. We collected this patient’s data until the end of cilostazol therapy until the operation and used them for the analysis. As part of the best medical treatment of PAD, all patients were taking 100 mg of aspirin daily and statins at the start of the study. Other medications of the participants were not changed.

At the first and then at every subsequent examination, we administered a supervised walking test for all patients with the help of a physiotherapist. We collected blood control samples from all patients before starting cilostazol therapy. After a general physical examination, participants rested for 30 min before peripheral blood samples were collected from the antecubital vein. Blood samples were taken from both the study group (n = 5 in each female and male category) and a healthy control group (n = 5 from both genders). The treatment regimen included administering 100 mg of cilostazol, which was taken twice a day. Patients attended clinical appointments after 2 weeks, 1 month, 2 months, and 3 months. At these check-ups, blood samples were collected, and walking distances of the patients were assessed. Throughout the follow-up period, no adverse reactions to cilostazol were observed. The study was approved by the Hungarian Medical Research Council (IV/2448-4/2022), and all participants signed a written statement of informed consent.

### 4.1. Blood Sample Preparation and Handling

Blood samples were drawn into specialized tubes containing EDTA (Vacuette™ EDTA Tube. Greiner Bio-One GmBH, Bad Haller Str. 32, 4550 Kremsmünster, Austria. EDTA: 1.5 mg/mL of blood). Red blood cells were isolated by centrifuging at 1500× *g* for 15 min at 4 °C to separate plasma from cellular components. Native human plasma samples were kept at 4 °C until DSC measurements were performed.

### 4.2. DSC Measurements

The thermal unfolding of the plasma was observed using a SETARAM Micro DSC-III calorimeter. We adhered to previously established methods [26]. The experiments were conducted over a temperature range of 0 to 100 °C, maintaining a steady heating rate of 0.3 °C/min. Denaturation studies were carried out using standard Hastelloy batch vessels containing an average sample volume of 950 µL, while a 0.9% NaCl solution served as the reference sample. Sample and reference equilibration were conducted with a precision of ±0.1 mg. A repeated scan of the denatured sample was subtracted from the original DSC curve to adjust the baseline. Heat flow was plotted as a function of temperature, and thermal parameters were determined by calculating the calorimetric enthalpy from the area under the heat flow curve within the 40–90 °C range. This calculation utilized the two-point peak integration feature of the SETSOFT software (Ref.: 09/49999 V1.4.0.). The thermal data are presented as averages ± standard deviations, with temperatures rounded to one decimal place and calorimetric enthalpy to two decimal places.

In the case of deconvolution, we have used the Origin 6.0 software (it is accepted worldwide, and its newer versions are from OriginLab, Northampton, MA, USA). To find the possible melting points of different protein compounds, we applied the values accepted by the literature. Gaussian curves were used during fitting. At the very first, the official denaturation temperatures were administrated, and after, the peaks, thickness, and area of Gaussian functions were changed manually, iterating the total area of Gaussians for the best fitting with the experimental curve. In addition to the R2 value, we accepted the deconvolution to be good when the difference between the total measured calorimetric enthalpy and the fitted one was in the range of the enthalpy detection range of our instrument (below 5%).

During thermal denaturation, melting temperature differences in the order of 1 °C are considered to be a notable change (in other words: if the differences are 2–3 times greater than the resolution power of the instrument, it is “significant” in thermoanalytical jargon).

## 5. Conclusions

This preliminary, proof-of-concept study provided novel insights into the biophysical alterations of plasma proteome in patients with intermittent claudication treated with cilostazol over a 3-month period. Using differential scanning calorimetry (DSC) combined with deconvolution analysis, we observed notable differences in thermal unfolding profiles between cilostazol-treated patients and healthy controls.

Although similar approaches have proven useful in oncology and inflammatory disease diagnostics [27,28,29], future studies with larger patient populations and extended follow-up periods are necessary to validate these preliminary observations, clarify underlying mechanisms, and assess whether DSC-derived plasma thermodynamic markers could serve as complementary biomarkers for monitoring disease progression and therapeutic response in intermittent claudication, especially in those cases where cilostazol is less or not effective within the 3-month treatment period.

This work serves as a proof-of-concept study requiring validation in larger cohorts before clinical implementation. Therefore, we plan to increase the sample size and extend the follow-up period to at least six months for valid statistical analysis and to provide middle-term results.

## Figures and Tables

**Table 1:** The characteristic thermodynamic parameters in the case of healthy controls and treated patients. *Tx* stands for the melting (denaturation or unfolding) temperature, Δ*H_Tcal_* is the average measured total calorimetric enthalpy normalized on sample mass with s.d. Data are rounded to one decimal place for temperatures and two decimal places for enthalpy. In the case of melting temperature and the contribution to the total enthalpy, s.d. is missing because the fitting was made from the average of scans. The **red bold** numbers refer to the definite deviation, referring to the start of the patient’s treatment. We have significant alterations referring to the healthy control and week 0 in all parameters.

Samples	*T*_1_ (°C) Fibrinogen	*T*_2_ (°C) Albumin	*T*_3_ (°C) Albumin, Globulins	*T*_4_ (°C) Globulins	*T*_5_ (°C) Globulins	*T*_6_ (°C) Transferrin	Δ*H_Tcal_* (Jg^−1^)
**Healthy**	56.0	63.0	68.0	72.0	76.0	82.5	1.29 ± 0.06
Area %	4.2	52.8	17.9	9.5	12.2	3.4	
**Week 0**	52.0	61.0	64.1	68.0	72.0	80.0	1.41 ± 0.11
Area %	4.6	13.5	40.0	13.2	20.7	9.4	
**Week 2**	**50.0**	61.2	63.6	68.7	**75.3**	**92.3**	1.41 ± 0.05
**Area %**	**6.0**	14.0	37.0	16.0	**14.6**	**4.6**	
**Week 4**	52.2	60.5	63.9	**69.5**	**78.0**	**90.7**	1.53 ± 0.24
**Area %**	**9.0**	**16.0**	36.3	**22.5**	**19.0**	**5.7**	
**Week 8**	52.0	**60.0**	63.7	68.6	**76.9**	**91.2**	1.48 ± 0.18
**Area %**	**7.5**	14.0	39.0	**26.5**	**18.5**	**5.2**	
**Week 12**	52.0	**59.1**	64.0	**70.8**	**80.0**	**92.0**	1.46 ± 0.08
**Area %**	**2.8**	**5.7**	43.0	**28.6**	**14.3**	**5.4**	

## Data Availability

The original contributions presented in the study are included in the article, further inquiries can be directed to the corresponding author.

## Abbreviations

The following abbreviations are used in this manuscript:

DSC	Differential scanning calorimetry
IC	Intermittent claudication
PAD	Peripheral artery disease
